# The Burden of Hospital Illness Associated with Disseminated Versus Isolated Pulmonary Coccidioidomycosis in the United States

**DOI:** 10.3390/jof11020161

**Published:** 2025-02-19

**Authors:** Craig I. Coleman, Jessica Bylyku, Andria Latifi, Belinda Lovelace, Ryan Shan, Lahar Miriyapalli, Fariba Donovan

**Affiliations:** 1 Department of Pharmacy Practice, University of Connecticut School of Pharmacy, Storrs, CT 06269, USA; jessica.bylyku@uconn.edu (J.B.); andria.latifi@uconn.edu (A.L.); ryan.shan@uconn.edu (R.S.); lahar.miriyapalli@uconn.edu (L.M.); 2 Health Economics and Outcomes Research, F2G, Inc., Princeton, NJ 08540, USA; belinda.lovelace@f2g.com; 3 The Valley Fever Center for Excellence, University of Arizona College of Medicine-Tucson, Tucson, AZ 85724, USA; faribadonovan@arizona.edu; 4 The Division of Infectious Diseases, Department of Medicine, University of Arizona College of Medicine-Tucson, Tucson, AZ 85724, USA; 5 BIO5 Institute, University of Arizona, Tucson, AZ 85721, USA

**Keywords:** coccidioidomycosis, cost of illness, pulmonary coccidioidomycosis, disseminated coccidioidomycosis, national inpatient sample

## Abstract

There are scarce data comparing inpatient mortality, length of stay (LOS) and all-cause hospital costs in disseminated coccidioidomycosis (DCM) vs. isolated pulmonary coccidioidomycosis (IPCM). We assessed the burden of hospital illness associated with DCM versus IPCM. This study was performed using National Inpatient Sample data from 2019 to 2021. DCM was defined as having a primary International Classification of Diseases—Tenth Revision (ICD-10) code for coccidioidal meningitis, a non-primary code for coccidioidal meningitis in the presence of a primary code for a meningitis complication or a procedure code depicting the need for a meningitis-related procedure, or a primary code for DCM without a code for unspecified disease. IPCM was defined as a primary code for pulmonary coccidioidomycosis without codes for DCM or unspecified disease. Multivariable regression was used to compare the odds of in-hospital mortality, LOS and all-cause hospital costs (2023 US$) for DCM versus IPCM, after covariate adjustment. A total of 6195 hospitalizations were identified, 2305 for DCM and 3890 for IPCM. Patients experiencing a DCM hospitalization had a 19.7% incidence of concomitant pulmonary coccidioidomycosis. Coccidioidal meningitis constituted 81.3% of all DCM hospitalizations, of which 78.1% received a meningitis-related procedure or were admitted for a meningitis complication. DCM was associated with an increased odds of death (odds ratio = 2.76, 95% confidence interval [CI] = 1.26–6.04) versus IPCM. DCM was associated with a longer mean hospital LOS (4.51 days, 95%CI = 3.39–5.63) and higher mean all-cause costs ($20,008, 95%CI = $15,313–$24,704) versus IPCM. DCM hospitalizations were associated with higher odds of inpatient mortality, longer LOS, and higher costs versus IPCM.

## 1. Introduction

*Coccidioides immitis* and *posadasii* are dimorphic fungi common to the western region of the United States (US) [1,2]. About 50,000 persons in the US become ill from coccidioidomycosis per year, with about 10,000 to 20,000 formally diagnosed [1,2,3]. Among coccidioidomycosis patients, prior research has shown that, during the course of their illness, about one-fourth will visit a healthcare provider > 10 times, nearly one-half will have more than one emergency department visit, and 41% will be hospitalized (median length of stay of 6 days; 25%, 75% range: 4, 10 days) [4].

Most coccidioidomycosis patients (~60%) have asymptomatic infections. Of symptomatic cases, many will develop isolated pulmonary (IPCM) disease, which may resolve spontaneously or after 3–6 months of antifungal treatment [1,2]. Disseminated coccidioidomycosis (DCM), which can affect the skin, bones, joints and central nervous system, may occur in ~0.5 to 2% of cases, and results in more widespread and severe symptoms and the need for a more aggressive and prolonged treatment [1,2,3].

There is a paucity of data comparing and quantifying differences in inpatient mortality, length of stay and all-cause hospital costs in DCM versus IPCM. We assessed the burden of hospital illness associated with DCM versus IPCM.

## 2. Materials and Methods

### 2.1. Study Population

We performed a retrospective study using National Inpatient Sample data from 2019 to 2021. The National Inpatient Sample database comprises hospital discharges, and was developed by the Healthcare Cost and Utilization Project and sponsored by the Agency for Healthcare Research and Quality [5]. The National Inpatient Sample is a 20% stratified systematic randomized sampling of all hospital discharges, drawn from states participating in the Healthcare Cost and Utilization Project (including all states in which coccidioidomycosis is considered endemic), and covering more than 97% of the US population, with all payer types. National Inpatient Sample data are compiled using a complex survey design^5^, requiring the use of survey-specific analysis methods that simultaneously account for clustering and stratification and the potential for sampling bias. Using these methods allows the weighting of hospital stays to generate nationally representative estimates with accompanying variance estimates [6]. The data were accessed in compliance with the Health Insurance Portability and Accountability Act (HIPAA). Institutional review board approval was not required for this analysis of de-identified secondary data.

Inclusion criteria for this study consisted of hospitalizations in adults (≥18 years) with a diagnosis of DCM or IPCM. DCM was defined by a primary International Classification of Diseases—Tenth Revision (ICD-10) diagnosis code for coccidioidal meningitis, a non-primary code for coccidioidal meningitis in the presence a primary diagnosis code for a common meningitis complication (i.e., hydrocephalus, cerebral vascular accident, encephalitis, encephalopathy, cerebral vasculitis or vasospasm, cerebral abscess, shunt complication, infusion device failure) or a procedure code depicting the need for a coccidioidal meningitis-related procedure (i.e., ventricular shunt, drain or infusion device placement, revision or removal), or a primary code for DCM without a code for unspecified coccidioidomycosis. IPCM was defined as a primary code for pulmonary coccidioidomycosis without codes for DCM (including evidence of unspecified central nervous system disease) or unspecified disease. Outcomes included all-cause inpatient mortality, hospital length of stay and all-cause total hospital treatment costs.

### 2.2. Statistical Analysis

We performed descriptive analysis on the weighted, nationally representative set of DCM and IPCM hospitalizations, and we report frequencies and means with 95% confidence intervals (CIs) for demographics, comorbidities and outcomes of interest for the overall combined and individual DCM and IPCM populations. Multivariable logistic regression was performed to compare the odds of in-hospital mortality for DCM versus IPCM hospitalizations, after adjustment for covariates. Multivariable general linear models were used to compare mean differences in length of stay and all-cause total costs between DCM and IPCM hospitalizations. Covariates included in the multivariable models are provided in Table 1. All charges were converted to costs using cost-to-charge ratios supplied by the Healthcare Cost and Utilization Project. Costs were inflated to 2023 US dollars based on the Consumer Price Index for Medical Care, obtained from the Bureau of Labor Statistics [7]. We performed a sensitivity analysis that compared only the coccidioidal meningitis subset of DCM to IPCM hospitalizations. Database management and statistical analysis was performed using IBM SPSS Statistics, version 28.0.1.1 (Armonk, New York, NY, USA).

## 3. Results

### 3.1. Patient Cohort

From 2019 to 2021, there were 28,915 weighted hospitalizations with at least one coccidioidomycosis ICD-10 code in any billing position. Of these, 2305 had DCM (1875 with coccidioidal meningitis) and 3890 had IPCM. Hospitalizations not meeting these criteria (i.e., non-DCM patients, those lacking a primary diagnosis code for pulmonary coccidioidomycosis, unspecified coccidioidomycosis) were excluded. The mean age for patients experiencing hospital stays was 50.8 years, with men making up 63.8% of the cohort. A plurality of those hospitalized with DCM or IPCM were of white race (43.3%), followed by Hispanic (31.8%), Black (11.6%) and Asian–Pacific (6.4%). Greater than 90% of hospitalizations occurred in the West US census region. The most frequent (>10%) comorbidities among the hospitalized patients present were chronic obstructive pulmonary disease or asthma, diabetes mellitus, drug or alcohol abuse, immunocompromised status and obesity. A diagnosis code for sepsis was present in 6.7% of all hospitalizations.

### 3.2. Unadjusted Outcomes

For the overall combined cohort of DCM and IPCM patients, the incidence of all-cause inpatient mortality was 3.1% (95%CI, 2.3 to 4.2), the mean hospital length of stay was 8.09 days (95%CI, 7.43 to 8.76) and the mean all-cause total costs were $28,917 (95%CI, $26,192 to $31,642) (Table 2). Prior to multivariable regression analysis, mortality, hospital length of stay and total costs were significantly higher for DCM versus IPCM hospitalizations (*p* < 0.001 for all).

### 3.3. Multivariable Analysis

Following multivariable logistic regression, DCM hospitalizations were associated with an increased odds of all-cause inpatient mortality (odds ratio, 2.76; 95%CI, 1.26 to 6.04) compared to IPCM hospitalizations (Table 3). Upon multivariable linear regression, DCM hospital stays appeared to be longer, on average, (delta (Δ) 4.51 days; 95%CI, 3.39 to 5.63) versus IPCM stays, and were associated with higher treatment costs (Δ $20,008; 95%CI, 15,313 to 24,704). Other covariates found to be predictors of an increased odds of all-cause inpatient mortality included age, Black race and sepsis. Predicators of longer length of stay included Black race, immunocompromised status and sepsis. Asian race and sepsis were significantly associated with increased all-cause treatment costs.

Coccidioidal meningitis constituted 81.3% of all DCM hospitalizations, with 52.8% having a primary diagnostic code, and 78.1% having other evidence of coccidioidal meningitis, including a procedure for meningitis management or a primary billing code for a meningitis complication. When the coccidioidal meningitis-only subset of DCM hospitalizations was compared to IPCM hospitalizations using multivariable regression, coccidioidal meningitis was associated with increased mortality (odds ratio 2.97; 95%CI, 1.29 to 6.86), longer length of stay (Δ 4.90 days; 95%CI, 3.66 to 6.13) and higher mean all-cause costs (Δ $22,050; 95%CI, $16,835 to $27,265) (Table 4).

## 4. Discussion

Our study, using the National Inpatient Sample data set, identified nationally representative samples of both DCM and IPCM hospitalizations from 2019 to 2021. Both DCM and IPCM hospitalizations were found to be prevalent in the National Inpatient Sample, with >2300 hospitalizations observed in each cohort. Analysis of these demonstrated that DCM hospitalizations were nearly 2.8-fold more likely to result in death, associated with greater than or equal to four additional days length of stay and costing > $20,000 more than an IPCM stay. Of all DCM hospitalizations observed, approximately four out of five were coded for coccidioidal meningitis. Given the substantial portion of coccidioidal meningitis hospitalizations, the differences in outcomes in the coccidioidal meningitis subgroup were similar to the main analysis results of DCM versus IPCM hospitalizations.

Prior studies evaluating the burden of illness associated with coccidioidomycosis have been published [8,9,10,11,12,13,14,15], most of which combine all coccidioidomycosis types as a single entity, rather than separately evaluating individual manifestations (i.e., pulmonary, disseminated, meningeal), or the studies focus on only a single endemic area [14,15]. We previously published a national burden of illness analysis of coccidioidal meningitis patients alone that quantified the substantial burden on hospital resources of this coccidioidomycosis manifestation [i.e., frequent meningitis-related procedures (63.6%), long length of stay (mean of 13 days) and high costs (mean of $48,155)] [13]. The present study evaluated separate cohorts of patients with disseminated disease or isolated pulmonary coccidioidomycosis, and, thus, provides valuable new insights into the hospital burden of these manifestations. While a recent study by Benedict and colleagues [16] evaluated outcomes within primary pulmonary coccidioidomycosis patients, this study did not assess the related burden of illness on hospitals. To our knowledge, the present study is the first to compare the outcomes and costs of disseminated and isolated pulmonary coccidioidomycosis from a hospital perspective.

The increased burden of illness associated with DCM and coccidioidal meningitis likely stems from the greater disease severity and complications in these patients, particularly those admitted for coccidioidal meningitis who frequently require more surgical intervention [2]. In a study by Sivasubramanian and colleagues [17] evaluating 133 patients with coccidioidal meningitis, 72% required surgical management (90% received a ventricular shunt) during a median follow-up of 36 months. In addition, 52.5% of these required a surgical revision of their shunt anywhere from one to six times (mean, 2.6), resulting in frequent rehospitalizations (about three rehospitalizations per patient). Notably, in our study of prevalent coccidioidomycosis patients captured at any time during their disease process, evidence of the need for a coccidioidal meningitis-related procedure (i.e., ventricular shunt, drain or infusion device placement, revision or removal) was seen in 78.1% of DCM hospitalizations.

Direct lifetime costs of treating all forms of coccidioidomycosis have been estimated to be as high as $64,800 per person diagnosed (2019 US$), with an additional $6300 in indirect (lost productivity/wages) costs [15]. DCM infection has the highest economic burden, at $1.26 million in direct costs and $137,400 in indirect costs per person, whereas primary uncomplicated coccidioidomycosis pneumonia costs $23,200 in direct costs and $1300 in indirect costs per person. Given the high burden of illness associated with DCM, there is an urgent need for novel antifungal treatments to manage disseminated manifestations.

Coccidioidomycosis is often misdiagnosed, or the diagnosis is delayed (median delay of 23 days), because it is difficult to distinguish from other respiratory diseases based on signs and symptoms alone [18]. In a surveillance study of 186 coccidioidomycosis patients performed by Benedict and colleagues [19], it was found that 70% of coccidioidomycosis patients were initially misdiagnosed (of whom 83% were prescribed antibacterials), and 54% or patients visited a healthcare provider > 3 times before being tested for coccidioidomycosis. Moreover, it is likely that patients with less severe cases of coccidioidomycosis go unreported or undiagnosed. These data suggest that there is a need to enhance awareness of coccidioidomycosis among clinicians, particularly given the high costs and mortality rates of DCM [18,19].

This study has limitations worth noting. First, no validation studies assessing the sensitivity and specificity of billing codes for coccidioidomycosis have been published. We used billing codes that are commonly used in analyses performed by the Centers for Disease Control and Prevention to identify DCM, coccidioidal meningitis and IPCM^1^ [20], as well as de novo supplemental ICD-10 diagnosis and procedural code algorithms. Despite these steps, misclassification due to miscoding cannot be ruled out [21]. In addition, due to a lack of detail in current ICD-10 billing codes for coccidioidomycosis (specifically a lack of billing codes for coccidioidomycosis associated with common manifestations of disseminated disease, such as bone, joint or skin complications), we were unable to further subset DCM hospitalizations, other than for coccidioidal meningitis. Studies attempting to validate ICD-10 billing code algorithms to identify coccidioidomycosis and specific disease phenotypes are needed. A second limitation is that we were unable to determine for how long patients had had coccidioidomycosis prior to hospitalizations. Thirdly, the cause of death is not distinguishable in the National Inpatient Sample database, and the data set only reports data through hospital discharge. Consequently, we could only report on all-cause inpatient mortality. Notably, the study data used were obtained during the height of the COVID-19 pandemic, which could have impacted our results [22]. Finally, the National Inpatient Sample database does not contain inpatient or outpatient medication data; therefore, we cannot comment on the usage, treatment patterns or appropriateness of antifungal treatment prior to or during the hospital stay.

DCM hospitalizations, including those for coccidioidal meningitis, were associated with significantly higher odds of inpatient mortality, a longer length of stay and higher costs versus IPCM.

## Figures and Tables

**Table 1:** Demographics, comorbidities and unadjusted outcomes of patients with disseminated or isolated pulmonary coccidioidomycosis.

	All N = 6195 %, (95%CI)	Disseminated N = 2305 %, (95%CI)	Isolated Pulmonary N = 3890 %, (95%CI)
**Demographics**			
Age (years, mean, 95%CI)	50.8 (49.7, 52.0)	46.9 (45.3, 48.4)	53.2 (51.6, 54.8)
Sex			
Women	36.2 (33.5, 39.1)	27.8 (23.7, 32.2)	41.3 (37.6, 45.1)
Men	63.8 (60.9, 66.5)	72.2 (67.8, 76.3)	58.7 (54.9, 62.4)
Race			
White	43.3 (39.9, 47.0)	30.4 (25.9, 35.2)	51.2(46.8, 55.5)
Black	11.6 (9.8, 13.8)	18.2 (15.0, 22.0)	7.7 (5.9, 10.1)
Hispanic	31.8 (28.7, 35.1)	34.7 (30.3, 39.4)	30.1 (26.1, 34.4)
Asian–Pacific	6.4 (5.1, 7.9)	9.3 (6.8, 12.6)	4.6 (3.4, 6.3)
Other	6.8 (5.5, 8.3)	7.4 (5.3, 10.2)	6.4 (4.9, 8.4)
Region ^‖^			
West	91.6 (90.7, 92.4)	89.2 (86.7, 91.2)	93.1 (91.7, 94.2)
Other	8.4 (7.6, 9.3)	10.8 (8.8, 13.3)	6.9 (5.8, 8.3)
Payer			
Medicare	27.6 (24.7, 30.8)	23.4 (19.9, 27.3)	30.1 (26.0, 34.5)
Medicaid	35.4 (32.3, 38.7)	42.1 (37.3, 47.1)	31.5 (27.9, 35.3)
Commercial/Private	29.5 (26.7, 32.6)	25.4 (21.3, 30.0)	32.0 (28.5, 35.7)
Other	7.4 (6.1, 9.0)	9.1 (7.0, 11.8)	6.4 (5.0, 8.3)
Year			
2019	34.9 (28.7, 41.8)	34.3 (27.0, 42.4)	35.3 (28.1, 43.3)
2020	32.2 (25.8, 39.3)	29.9 (23.0, 37.9)	33.5 (26.3, 41.7)
2021	32.8 (26.6, 39.8)	35.8 (28.2, 44.1)	31.1 (24.5, 38.6)
**Comorbidities**			
COPD or asthma	16.6 (14.5, 19.0)	10.6 (8.0, 14.1)	20.2 (17.4, 23.2)
COVID-19	1.3 (0.7, 2.2)	1.7 (0.9, 3.4)	1.0 (0.5, 2.0)
Diabetes mellitus	33.5 (31.1, 36.0)	24.7 (21.0, 28.9)	38.7 (35.2, 42.3)
Drug or alcohol Abuse	9.9 (8.3, 11.8)	11.1 (8.6, 14.1)	9.3 (7.4, 11.5)
Immunocompromised ^‡^	10.7 (9.0, 12.6)	13.7 (10.8, 17.1)	8.9 (7.1, 11.1)
Obesity	12.9 (11.0, 15.1)	8.0 (5.8, 10.9)	15.8 (13.3, 18.7)
Sepsis	6.7 (5.5, 8.2)	13.9 (11.2, 17.1)	2.4 (1.6, 3.7)
**Coccidioidomycosis Presentation**			
Concomitant pulmonary disease	--	19.7 (16.3, 23.7)	--
Coccidioidal meningitis	--	81.3 (77.6, 84.6)	--
Primary diagnosis code	--	52.8 (47.9, 57.7)	--
Coccidioidal meningitis-related procedure *	--	78.1 (74.0, 81.8)	--
Meningitis complication †	--	28.0 (23.3, 33.2)	--

CI = confidence interval, COPD = chronic obstructive pulmonary disease. † Primary diagnosis code for common meningitis complication, including hydrocephalus, cerebral vascular accident, encephalitis, encephalopathy, cerebral vasculitis or vasospasm, cerebral abscess, shunt complication or infusion device failure. * Includes ventricular shunt, drain or infusion device placement, revision or removal. ^‡^ Defined as presence of human immunodeficiency virus, autoimmune diseases, myelodysplastic syndrome, neutropenia, history or complication of transplant, or immunosuppressive disorders. ^‖^ West was defined per US census region; Other included Northeast, Midwest and South census regions.

**Table 2:** Unadjusted outcomes of patients with disseminated or isolated pulmonary coccidioidomycosis.

	All N = 6195 %, (95%CI)	Disseminated N = 2305 %, (95%CI)	Isolated Pulmonary N = 3890 %, (95%CI)
Unadjusted Outcomes			
Death (%)	3.1 (2.3, 4.2)	6.1 (4.3, 8.5)	1.4 (0.8, 2.5)
Length of stay (days, mean, 95%CI)	8.09 (7.43, 8.76)	12.26 (10.83, 13.69)	5.63 (5.23, 6.03)
Total costs (2023 US$, mean, 95%CI)	$28,917 (26,192, 31,642)	$46,960 (41,271, 52,648)	$18,250 (16,335, 20,164)

**Table 3:** Predictors of all-cause inpatient mortality, hospital length of stay and all-cause total hospital costs following multivariate regression analysis in patients with disseminated coccidioidomycosis or isolated pulmonary coccidioidomycosis.

Covariates	Mortality * OR (95%CI)	Length of Stay * Mean, Δ, Days (95%CI)	Treatment Costs * Mean, Δ, US$ (95%CI)
**Coccidioidomycosis Presentation**			
Isolated pulmonary	Referent	Referent	Referent
Disseminated	2.76 (1.26, 6.04)	4.51 (3.39, 5.63)	$20,008 (15,313, 24,704)
**Demographics**			
Age (increase per year)	1.04 (1.01, 1.07)	0.002 (−0.04, 0.05)	$1 (−169, 171)
Sex			
Women	Referent	Referent	Referent
Men	0.94 (0.44, 2.04)	0.78 (−0.17, 1.73)	$2695 (−1288, 6677)
Race			
White	Referent	Referent	Referent
Black	3.78 (1.40, 10.22)	2.22 (0.05, 4.39)	$4828 (−2069, 11,725)
Hispanic	1.19 (0.40, 3.61)	0.16 (−0.93, 1.24)	$856 (−4027, 5739)
Asian	1.50 (0.29, 7.71)	3.32 (−0.51, 7.15)	$19,902 (5419, 34,384)
Other/unknown	1.18 (0.26, 5.47)	0.14 (−1.71, 1.98)	−$2725 (−11,057, 5608)
Hospital region			
West	Referent	Referent	Referent
Other	0.79 (0.26, 2.36)	1.13 (−0.96, 3.21)	−$5444 (−11,163, 275)
Payer			
Medicare	Referent	Referent	Referent
Medicaid	1.61 (0.53, 4.92)	−0.02 (−2.35, 2.31)	$2895 (−5113, 10,903)
Private	1.30 (0.47, 3.64)	−1.58 (−3.49, 0.32)	$1904 (−4838, 8646)
Other	0.56 (0.08, 4.02)	−1.57 (−3.91, 0.77)	−$1846 (−10,095, 6404)
Year			
2019	1.77 (0.67, 4.68)	0.43 (−0.92, 1.78)	$1189 (−3975, 6354)
2020	1.78 (0.64, 5.00)	0.23 (−1.24, 1.69)	$2314 (−3668, 8295)
2021	Referent	Referent	Referent
**Comorbidities**			
COPD or asthma			
No	Referent	Referent	Referent
Yes	1.74 (0.72, 4.20)	−0.51 (−1.93, 0.90)	$−1947 (−6821, 2926)
COVID-19			
No	Referent	Referent	Referent
Yes	2.58 (0.88, 75.57)	1.11 (−3.98, 6.20)	$34,715 (−9750, 79,180)
Diabetes mellitus			
No	Referent	Referent	Referent
Yes	1.15 (0.54, 2.45)	0.94 (−0.27, 2.15)	$4170 (−1050, 9390)
Drug or alcohol abuse			
No	Referent	Referent	Referent
Yes	1.28 (0.36, 4.57)	−2.16 (−1.93, 1.47)	−$2022 (−11,691, 7647)
Immunocompromised			
No	Referent	Referent	Referent
Yes	0.85 (0.29, 2.45)	2.10 (0.09, 4.11)	$2340 (−3512, 8192)
Obesity			
No	Referent	Referent	Referent
Yes	1.30 (0.44, 3.86)	−0.29 (−1.47, 0.90)	$3074 (−4770, 10,918)
Sepsis			
No	Referent	Referent	Referent
Yes	16.33 (7.65, 34.90)	13.09 (8.39, 17.78)	$64,270 (42,085, 86,454)

CI = confidence interval; COPD = chronic obstructive pulmonary disease; **Δ** = delta change; OR = odds ratio. * Covariates adjusted for included age, sex, race, region of hospital location, payer type, year of hospitalization, presence of chronic obstructive pulmonary disease (COPD)/asthma, COVID-19, diabetes, drug/alcohol abuse, immunocompromised status, obesity and sepsis.

**Table 4:** Predictors of all-cause inpatient mortality, hospital length of stay, and all-cause total hospital costs following multivariate regression analysis in subgroup of disseminated coccidioidomycosis patients with coccidioidal meningitis only or isolated pulmonary coccidioidomycosis.

Covariates	Mortality * OR (95%CI)	Length of Stay * Mean, Δ, Days (95%CI)	Treatment Costs * Mean, Δ, US$ (95%CI)
**Coccidioidomycosis Presentation**			
Isolated pulmonary	Referent	Referent	Referent
Coccidioidal meningitis	2.97 (1.29, 6.86)	4.90 (3.66, 6.13)	$22,050 (16,835, 27,265)
**Demographics**			
Age (increase per year)	1.04 (1.00, 1.08)	0.0 (−0.05, 0.05)	$ 9 (−165, 183)
Sex			
Women	Referent	Referent	Referent
Men	1.13 (0.52, 2.46)	0.70 (−0.26, 1.66)	$2424 (−1709, 6556)
Race			
White	Referent	Referent	Referent
Black	3.39 (1.17, 9.79)	3.26 (0.69, 5.84)	$9428 (1534, 17,323)
Hispanic	0.95 (0.30, 3.05)	0.24 (−0.98, 1.46)	$1553 (−3643, 6749)
Asian	1.31 (0.23, 7.66)	3.42 (−0.42, 7.25)	$20,523 (6043, 35,003)
Other/unknown	1.36 (0.28, 6.64)	0.14 (−1.92, 2.20)	$−2305 (−11,463, 6854)
Hospital region			
West	Referent	Referent	Referent
Other	0.55 (0.18, 1.67)	−0.46 (−2.65, 1.74)	−$7903 (−13,798, −2009)
Payer			
Medicare	Referent	Referent	Referent
Medicaid	1.89 (0.62, 5.79)	−0.35 (−2.66, 1.96)	$2136 (−5962, 10,233)
Private	1.19 (0.37, 3.85)	−1.94 (−3.89, 0.00)	$1303 (−5768, 8373)
Other	0.65 (0.09, 4.85)	−2.05 (−4.52, 0.43)	−$3290 (−11,928, 5349)
Year			
2021	Referent	Referent	Referent
2020	2.09 (0.68, 6.46)	0.40 (−1.10, 1.90)	$3077 (−3255, 9409)
2019	2.07 (0.67, 6.44)	0.61 (−0.75, 1.97)	$1594 (−3934, 7121)
**Comorbidities**			
COPD or asthma			
No	Referent	Referent	Referent
Yes	2.02 (0.80, 5.06)	−0.28 (−1.76, 1.20)	−$1214 (−6355, 3926)
COVID-19			
No	Referent	Referent	Referent
Yes	3.72 (0.09, 153.05)	1.22 (−4.43, 6.86)	$35,948 (−14,160, 86,056)
Diabetes mellitus			
No	Referent	Referent	Referent
Yes	1.02 (0.49, 2.45)	0.94 (−0.36, 2.25)	$4180 (−1301, 9661)
Drug or alcohol abuse			
No	Referent	Referent	Referent
Yes	1.21 (0.23, 4.51)	−0.35 (−2.10, 1.40)	−$2511 (−12,441, 7418)
Immunocompromised			
No	Referent	Referent	Referent
Yes	0.90 (0.30, 2.67)	2.46 (0.29, 4.64)	$2631 (−3693, 8954)
Obesity			
No	Referent	Referent	Referent
Yes	1.72 (0.59, 5.04)	−0.05 (−1.25, 1.15)	$3916 (−4391, 12,223)
Sepsis			
No	Referent	Referent	Referent
Yes	17.02 (7.48, 38.73)	13.43 (8.50, 18.37)	$64,434 (40,923, 87,944)

CI = confidence interval, COPD = chronic obstructive pulmonary disease; **Δ** = delta change; OR = odds ratio. * Covariates adjusted for included age, sex, race, region of hospital location, payer type, year of hospitalization, presence of chronic obstructive pulmonary disease (COPD)/asthma, COVID-19, diabetes, drug/alcohol abuse, immunocompromised status, obesity and sepsis.

## Data Availability

The original contributions presented in this study are included in the article. Further inquiries can be directed to the corresponding author.
